# Aging and caries experience in adults: a hospital-based cross-sectional study

**DOI:** 10.3389/fdmed.2026.1859510

**Published:** 2026-06-01

**Authors:** Manjit Talwar, Ali Borzabadi-Farahani, Monika Kamboj, Priyanka Gupta, Sapna Singla, Suresh Sharma

**Affiliations:** 1 Department of Dentistry, Government Medical College & Hospital, Chandigarh, India; 2 Clinical Reader, Department of Orthodontics, School of Dentistry, Cardiff University, Cardiff, United Kingdom; 3 Department of Statistics, Panjab University, Chandigarh, India

**Keywords:** adult caries experience, aging and caries experience, cross-sectional study, DMFT, root caries

## Abstract

**Background:**

A cross-sectional study conducted from April 2016 to April 2018 at the Government Medical College and Hospital in Chandigarh, India, investigated coronal and root caries prevalence and DMFT (Decayed, Missing, Filled Teeth) indices among urban, middle-income adults.

**Methodology:**

Overall, 601 individuals examined, aged 21–50 years, divided into three age groups: 21–30 (G1, *n* = 200), 31–40 (G2, *n* = 201), and 41–50 (G3, *n* = 200). Using WHO Oral Health Assessment criteria, a calibrated examiner assessed caries; data graphically analysed and with Kruskal–Wallis tests with pairwise tests (3 age group comparisons), independent samples Kruskal–Wallis test (male/female comparison), and Spearman's correlations.

**Results:**

The overall mean DMFT score was 6.52 (95% CI, 6.16–6.88), rising from 5.12 in G1 to 8.12 in G3 (*P* < 0.01). DMFT component scores were D = 4.02, M = 1.73, and F = 0.77. Age-related increase in decayed teeth prevalence (86% in G1, 86.5% in G2, 89.5% in G3), missing teeth (36%, 54.5%, 73.5%), and filled teeth (25%, 35.5%, 35.5%) was observed. Root caries prevalence was low (3%–4.5%), with no significant age-related differences (*P* > 0.05). Females had significantly higher mean rank for DMFT, decayed, missing, and filled teeth scores than males (*P* < 0.05), but no gender differences were found for root caries (*p* = 0.195). Age weakly correlated with DMFT (r = 0.262, *P* < 0.001) and filled teeth (r = 0.093, *P* = 0.023), but not with decayed teeth or root caries.

**Conclusion:**

The study highlights the need for early preventive interventions, especially for younger adults and females, to curb caries progression, though weak correlations limit clinical significance.

## Introduction

Dental caries remains a significant public health concern globally, contributing to tooth loss and reduced quality of life in adults and older adults. In India, the burden of dental caries and periodontal disease is increasing among adults. Several studies have assessed the prevalence of oral diseases in elderly populations across different regions of the country (1–7). Early intervention in appropriate adult age groups can prevent these conditions. Older adults, defined as individuals aged 65 years and above (young-old: 65–74 years, old-old: 75–84 years, oldest-old: 85 years and above), often have comorbidities, and poor oral health can exacerbate systemic health issues, further impacting quality of life (8–10).

With the growing population of older adults in India, preserving the oral health of young adults is a pressing need. Implementing preventive oral health measures early in adulthood is essential to ensure good oral health in the future aging population. To achieve better oral health outcomes, understanding the current oral disease status and developing management strategies for young adults (aged 20 years and above) are critical. Few studies in India have investigated the prevalence of dental caries in young adults (11–14). For instance, Athuluru et al. (15) reported a mean DMFT (Decayed, Missing, Filled Teeth) of 3.24 in the 35–44-year age group in an epidemiological study conducted in Nellore, Andhra Pradesh. To our knowledge, no studies in India have compared dental caries prevalence across various adult age groups.

The primary aim of this study was to examine the prevalence of dental caries, root caries, and DMFT scores in three adult age groups (21–30, 31–40, and 41–50 years). The secondary aim was to compare coronal and root caries across these groups, evaluate the effects of age and gender on dental caries, and explore the relationship between coronal (DMFT) and root caries experience and participants’ age.

## Materials and methods

### Study design and duration

A cross-sectional study was conducted among patients attending the outpatient Department of Dentistry at Government Medical College and Hospital, Chandigarh, India. Data were collected from April 2016 to April 2018.

### Ethical approval and consent

The study protocol was approved by the Ethics Committee of XXXX XXXX, XXXX (Reference No. GMCH/ETHICS/2016-110781, dated April 6, 2016). All participants received a patient information sheet and provided written informed consent before enrolment. All the study was conducted in the accordance with relevant or the institutional guidelines (Government Medical College & Hospital, Sector-32, Chandigarh, Pin 160031, India).

### Sample size

The sample size was calculated using the formula:

$$n=Z^{2}\times p\times (1-p/m^{2}),$$

where *Z* = 1.96 (for a 95% confidence level), *p* = 0.72 (anticipated prevalence), 1 - *p* = 0.28, and *m* = 0.05 (relative precision). Assuming a 95% confidence level and 5% relative precision, the calculated sample size was 596 (16). A total of 601 participants were enrolled to account for potential dropouts.

### Study population and setting

Participants were newly registered outpatients at the Department of Dentistry who met the inclusion and exclusion criteria.

#### Inclusion criteria

Participants aged 21–50 years who had completed 21, 31, or 41 years of age and reached 30, 40, or 50 years were included. All were urban residents of the Tricity area (Chandigarh, Mohali, Panchkula), belonged to the middle-income group (monthly salary above INR 25,000), and reported brushing their teeth once daily.

#### Exclusion criteria

Individuals with mental or physical disabilities, systemic diseases, or those taking medications were excluded, as were non-residents of the Tricity area and those who did not provide consent. The sample was stratified by age into three groups [age groups: 21–30 (*n* = 200), 31–40 (*n* = 201), and 41–50 (*n* = 200) years]: Group I (21–30 years; 103 males, 97 females), Group II (31–40 years; 75 males, 126 females), and Group III (41–50 years; 79 males, 121 females).

### Caries experience (DMFT, non-radiographic assessment)

DMFT (Decayed, Missing, Filled Teeth) was recorded using the WHO Oral Health Assessment Form (2013) (16). Coronal and root caries were assessed using a mouth mirror and CPI probe (16). A single calibrated examiner (MK) conducted all assessments (kappa score >0.84 in two consecutive tests two weeks apart). To evaluate intra-examiner variability, 15% of recordings were repeated in each age group and found to be consistent. No radiographic examinations were performed.

### Statistical analysis

Data were analysed using IBM SPSS Statistics version 23.0 (Chicago, IL, USA). Data normality was assessed using Q–Q plots and the Kolmogorov–Smirnov and Shapiro–Wilk tests, which confirmed a non-normal distribution.

Descriptive statistics, including the prevalence of decayed, missing, and filled teeth, were calculated. The mean DMFT (standard deviation, SD) for coronal and root caries was computed for each age group.

Nonparametric tests (Kruskal–Wallis test) were used to compare DMFT, decayed, missing, and filled teeth, as well as root caries, across age groups and genders.

Scatter plots and Spearman's rank correlation coefficients (r) were used to assess the relationships between participants’ age and their DMFT scores, as well as its individual components (DT, FT, and MT). Histograms, bar charts, and error bars were used to illustrate the distribution of DMFT and root caries scores in the study sample. A *p*-value < 0.05 was considered statistically significant.

## Results

The prevalence of coronal dental caries ranged from 86.0% to 89.5% across the study population: 86.0% in Group I (21–30 years), 86.5% in Group II (31–40 years), and 89.5% in Group III (41–50 years) (Table 1). A similar trend was observed for missing teeth (36.0%, 54.5%, 73.5%) and filled teeth (25.0%, 35.5%, 35.5%) across these groups.

**Table 1:** Prevalence of decayed, missing, and filled teeth across three age groups and by sex, including mean (SD) and 95% confidence intervals (CI) for DMFT, DT, FT, MT, and root caries in the sample.

Groups	Age Group	Decayed Teeth (DT)			Missing Teeth (MT)			Filled Teeth (FT)		
		Total	Gender		Total	Gender		Total	Gender	
			Male	Female		Male	Female		Male	Female
I	21–30 years	86%	84.5%	87.6%	36%	31.1%	41.2%	25%	24.3%	25.8%
II	31–40 years	86.5%	82.7%	88.1%	54.5%	48.0%	58.4%	35.5%	24.0%	42.6%
III	41–50 years	89.5%	87.3%	90.9%	73.5%	68.4%	76.9%	35.5%	36.7%	34.7%

Variable/Sex	DMFT (Mean (SD), 95% CI)	DT (Mean (SD), 95% CI)	MT (Mean (SD), 95% CI)	FT (Mean (SD), 95% CI)	Root caries (Mean (SD), 95% CI)
Male	5.49 (4.03) (4.99–5.98)	3.56 (3.37) (3.15–3.97)	1.35 (2.13) (1.08–1.61)	0.58 (1.24) (0.43–0.73)	0.051 (0.26) (0.02–0.09)
Female	7.29 (4.66) (6.80–7.79)	4.37 (3.22) (4.02–4.71)	2.01 (2.76) (1.72–2.30)	0.92 (1.76) (0.73–1.10)	0.055 (0.36) (0.017–0.083)
Independent Samples Kruskal–Wallis tests	*p* < 0.001	*p* < 0.001	*p* < 0.001	*p* = 0.04	*p* = 0.195

The prevalence of root caries was low, ranging from 3.0% to 4.5%: 3.5% in Group I, 3.0% in Group II, and 4.5% in Group III (Table 2). The overall mean DMFT score was 6.52 (SD = 4.49; 95% CI, 6.16–6.88). Mean DMFT (SD) values for coronal caries were 5.12 (3.56) in Group I, 6.32 (4.19) in Group II, and 8.12 (5.08) in Group III, showing an age-related increase in caries prevalence (Table 1).

**Table 2:** Mean (SD), median, and 95% confidence intervals (CI) for DMFT, root caries, coronal caries (D), missing teeth (M), and filled teeth (F) across three age groups.

DMFT, Groups	Age in years	N	Mean (SD)	95% CI for mean	Median
I	21–30	200	5.12 (3.56)	4.62–5.61	5
II	31–40	201	6.32 (4.19)	5.74–6.91	6
III	41–50	200	8.12 (5.08)	7.41–8.83	8

Root Caries, Groups	Age in years	N	Mean (SD)	95% CI for mean	
I	21–30	200	0.070 (0.38)	0.016–0.123	0
II	31–40	201	0.049 (0.26)	0.013–0.086	0
III	41–50	200	0.11 (0.34)	0.062–0.158	0

Coronal Caries (D), Groups	Age in years	N	Mean (SD)	95% CI for mean	
I	21–30	200	3.72 (3.06)	3.29–4.15	3
II	31–40	201	4.05 (3.41)	3.57–4.53	3
III	41–50	200	4.3 (3.43)	3.82–4.77	4

Missing Teeth (M), Groups	Age in years	N	Mean (SD)	95% CI for mean	
I	21–30	200	0.79 (1.38)	0.59–0.98	0
II	31–40	201	1.42 (2.04)	1.13–1.70	1
III	41–50	200	2.98 (3.27)	2.52–3.43	2

Filled teeth (F), Groups	Age in years	N	Mean (SD)	95% CI for mean	
I	21–30	200	0.61 (1.48)	0.40–0.82	0
II	31–40	201	0.86 (1.63)	0.63–1.08	0
III	41–50	200	0.85 (1.58)	0.63–1.07	0

Kruskal–Wallis tests confirmed no significant differences in the mean ranks of coronal caries (D) among the three age groups (*P* = 0.275). However, the tests provided strong evidence of differences in the mean ranks between at least one pair of age groups for the DMFT score (*p* < 0.001; all three age groups differed), missing teeth (M) (*p* < 0.001; all three age groups differed), teeth with fillings (F) (*p* = 0.038), and root caries (*p* = 0.024; only groups 2 and 3 differed).

**Figure 1:**
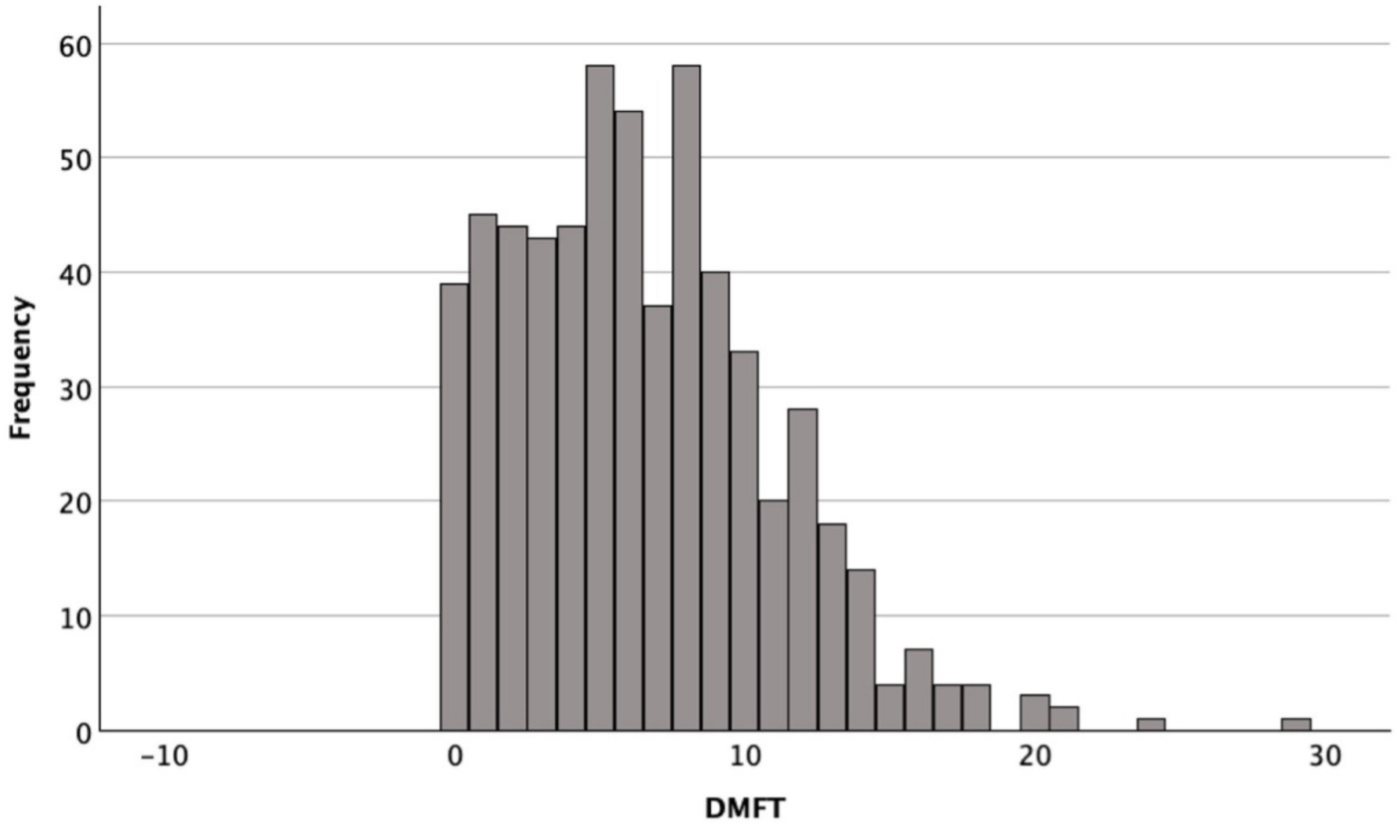
Distribution of DMFT scores in the study sample (*n* = 601).

The mean (SD) scores for the DMFT components were: D = 4.02 (3.31), M = 1.73 (2.53), and F = 0.77 (1.57). The mean root caries score was 0.053 (SD = 0.32, 95% CI, 0.03-0.08). A histogram of DMFT scores for the sample is presented in Figure 1.

An independent-samples Kruskal–Wallis test revealed that males and females had significantly different mean ranks for DMFT (*p* < 0.001), DT (*p* < 0.001), MT (*p* < 0.001), and FT (*p* = 0.04) scores. However, no significant difference in mean rank was observed in root caries scores between males and females (*p* = 0.195) (Table 1).

For root caries, the mean (SD) number of teeth with root caries was 0.070 (0.38), in Group I, 0.049 (0.26), in Group II, and 0.11 (0.34) in Group III (Table 2).

The data showed an upward trend in root caries increasing with age (Table 2).

**Figure 2:**
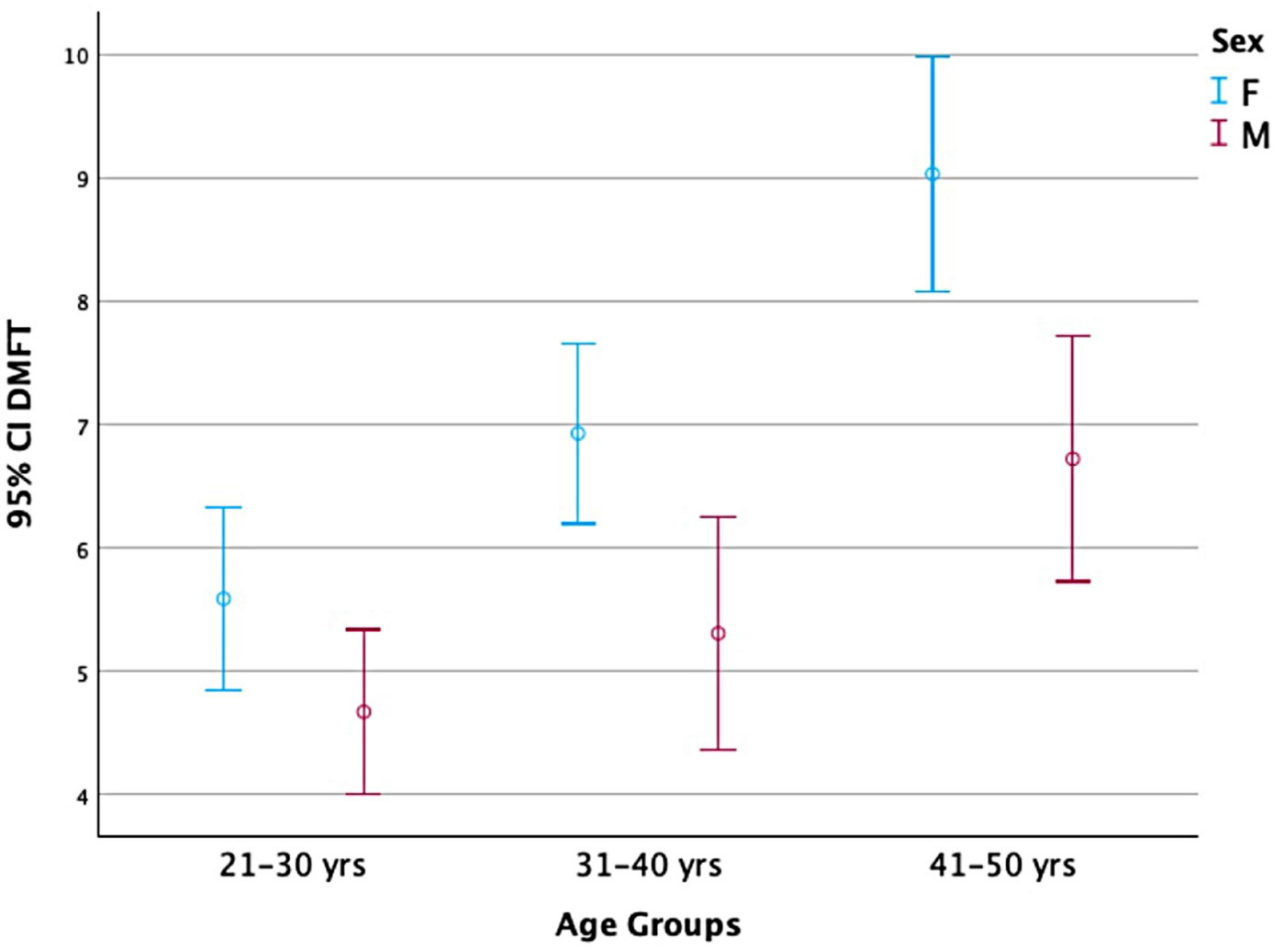
95% confidence intervals of mean DMFT scores for different age groups, stratified by sex.

**Figure 3:**
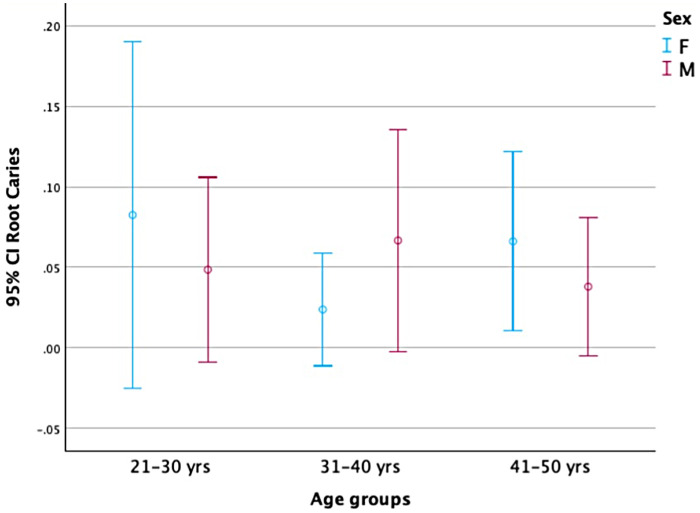
95% confidence intervals of mean root caries scores for different age groups, stratified by sex.

Gender-wise comparisons for DMFT were conducted using t-tests (Table 2). No significant difference was found in Group I (*P* > 0.05) and Group II (*P* > 0.05), but this was significant in Group III (*P* < 0.01), with females exhibiting higher mean DMFT than males in all groups (Figure 2). For root caries, t-tests showed no significant gender differences across all age groups (*p* > 0.05) (Figure 3).

**Figure 4:**
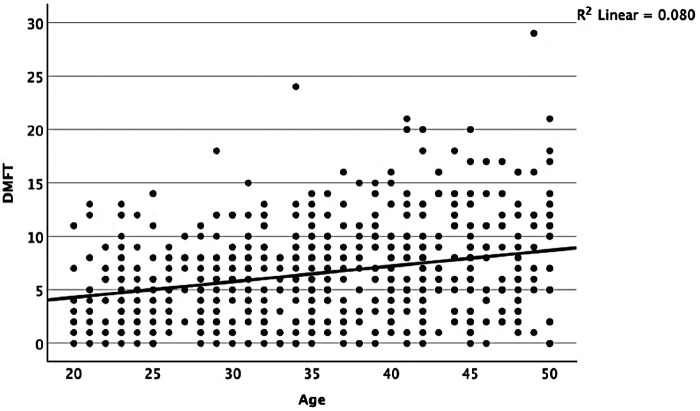
Scatter plot of participants’ DMFT scores (overall caries experience) vs. age (*n* = 601). The solid line indicates the regression trend.

**Figure 5:**
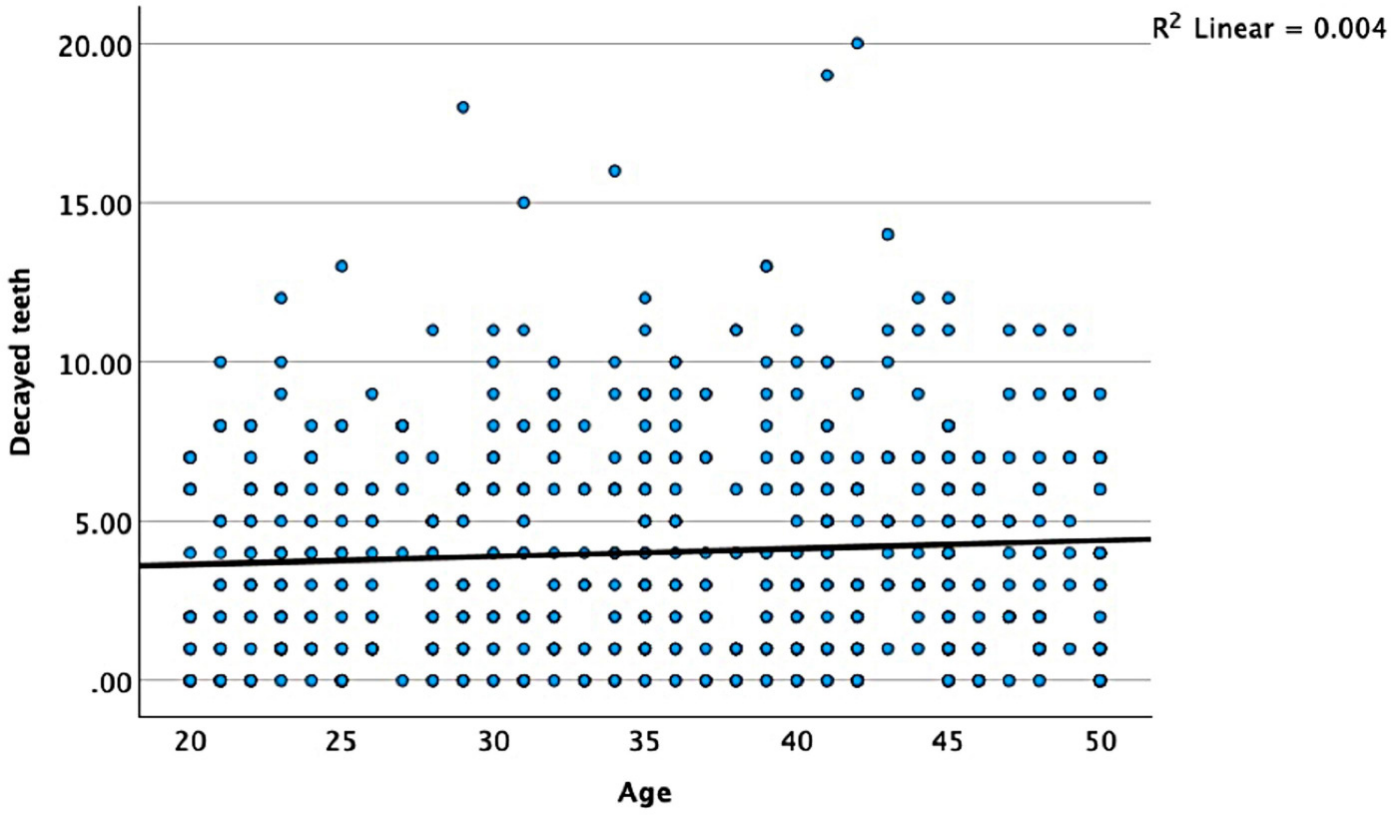
Scatter plot of participants’ DT scores (number of active carious teeth) vs. age (*n* = 601). The solid line indicates the regression trend.

**Figure 6:**
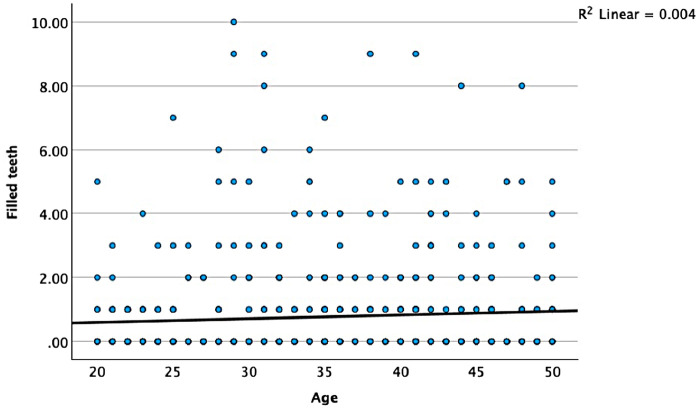
Scatter plot showing participants’ FT scores (number of filled teeth) in relation to age (*n* = 601). The solid line represents the regression trend.

Significant positive correlations were observed between participants’ age and both DMFT (r = 0.262, *p* < 0.001) and FT scores (r = 0.093, *p* = 0.023). However, no significant associations were found with DT (r = 0.062, *p* = 0.128) or root caries (r = 0.011, *p* = 0.769). Figures 4–6 show the relationship between the age of the participants and DMFT, DT and FT scores, respectively.

## Discussion

Findings of this cross-sectional study carried out in Chandigarh in a tertiary care hospital population of adults indicate that increasing age has an adverse impact on dental caries prevalence. The high prevalence of dental caries is suggestive of the need for early preventive interception. Studies carried out in India by various investigators among adults have reported a high burden of dental diseases with increasing age. Thomas et al. (1) carried out a study in South India and found a mean DMFT of 13.51 teeth in the 60-year and above age group. Patro et al. (17) reported similar findings from an urban resettlement colony of New Delhi in a cross-sectional study in the above 60-year age group and found a mean DMFT to be 13.8 teeth. Poorani and Chandana (18) reported a prevalence of 77.7% in the age group of 60 years and above in a Chennai population. They also observed that the percentage of females with dental caries was higher than males and DMFT was 12.01 in the 65–74-year age group. Shah et al. (19) in an oral health survey in a rural area of Faridabad, Haryana, found the prevalence of dental caries to be 64.9% and 50.1% in the 35–44- and 65–74-year age groups. Contradictory findings were reported in a study by Shetty et al. (20), where it was observed that the mean number of teeth affected by caries in <40 years and >40 years was 2.70 (1.98) and 2.83 (1.89), with not much difference with increasing age.

Most preventive programmes are targeted towards children. In a longitudinal study conducted by Broadbent et al. (21), it was reported that, even though preventive measures were provided in childhood and adolescence, dental caries was greater in adults. Similar findings have been reported by Bernabe and Sheiham (22). Dental caries is a major problem among adults that can be prevented, and its severity reduced (23, 24). It is important to have promotive and preventive oral health programmes for young adults (25). The prevalence of decayed and missing teeth is indicative of the urgent need for early preventive intervention in the 21–30-year age group in order to prevent dental caries and tooth loss over the years by conducting preventive educative programmes on oral hygiene and diet in young adults, who will constitute the future geriatric population. Continuous prevention is required, and preventive programmes should focus on all age groups. The study has limitations, as the population selected was from a tertiary care hospital and localized to a specific area of north India, so it is not generalizable to the entire country. The population was totally urban. A larger population sample should be studied with representation from rural and urban areas. More studies at the national level need to be conducted in the adult population.

A gender-wise comparison of males vs. females in all three age groups showed that females had higher mean DMFT scores compared to males in this urban population of Chandigarh. This difference was not significant in the 21–30-year age group (Group I) and the 31–40-year (Group II) but for the 41–50-year (Group III) group, females had statistically significantly higher DMFT scores for coronal caries. Root caries showed a similar trend; however, the root caries scores were low, and there was no statistically significant difference between males and females. The higher prevalence of dental caries in females may be attributed to factors like different salivary composition, flow rate, dietary habits, genetic variations, hormonal fluctuations, and particular social roles among their family (26). These factors need to be investigated further. Contradictory findings have been reported by Karuveettil et al. (27) in a meta-analysis exploration on gender being a risk factor for oral diseases in India, which reported dental caries to be higher in males compared to females, with a prevalence of 52% in males and 48% in females.

The percentage of decayed teeth increased from 86% (Group I) to 86.5% (Group II) to 89.5% (Group III), and missing teeth increased from 36% (Group I) to 54.5% (Group II) to 73.5% (Group III) with increasing age. This population, being urban and having better access to health services compared to other cities of the country, showed the filled component increased from 25% (Group I) to 35% (Group II) to 35.5% (Group III). Consistent with our observations, previous studies have reported that advancing age is accompanied by an upward trend in both caries prevalence and mean DMFT scores (28, 29).

Direct comparison of our results with earlier studies is difficult because of variations in their research methodologies; for instance, Jordan et al. (29) reported a DMFT score of 8.3 teeth for 35- to 44-year-old Germans. Another study (31) of 18–79-year-old Colombians reported a mean DMFT of 10.27 (7.11). In Saudi Arabia (32), DMFT scores were lowest in the 19–25 age group [9.33 (5.23)] and highest in the 36–40 group [13.42 (4.94)]. In an Indonesian population (33), the highest average DMFT scores were observed among respondents aged 45–64 years [10.97 (8.34)] and in those aged over 65 years [17.24 (10.52)].

According to a recent meta-analysis (34), the highest root caries prevalence was reported in Mexico (96.5%) (35), while the lowest was in Denmark (4%) (36). The highest root caries prevalence for North America was observed in Canada (19.7%) (37), for South America in Mexico (96.5%) (35), for Europe in Sweden (54%) (38), and for Asia in Sri Lanka (89.7%) (39). Jiang et al. (40) concluded that subjects who were older, female, with poorer oral and systemic health status, suboptimal oral hygiene behaviours, and lower oral health knowledge levels were more likely to be identified as a high-risk group for root caries. This can partially explain the very low prevalence (3%–4.5%) of root caries in the present sample, as subjects with physical disability, systemic disease, and those on medications were excluded from this study.

Previous research has consistently indicated that advancing age is linked to an elevated risk of root caries (40–43). Several investigations have also examined the influence of gender, though findings remain inconsistent regarding whether males or females are more affected (40, 44–47). In contrast, our analysis showed no statistically significant differences in root caries prevalence between genders within any of the three age categories, nor among the age groups themselves (*P* > 0.05).

We also examined the relationship between caries experience and participants’ age. According to Sheiham and Sabbah (48), caries levels in populations tend to follow predictable trend lines when environmental conditions remain stable and no effective interventions are implemented. Consistent with this, the present study found that mean DMFT scores were significantly higher in the 31–40 and 41–50 year age groups compared with the 21–30 year group. Scatter plots also demonstrated an upward trend in caries experience with increasing age. Similar findings have been reported in studies from northern India (49), southern India (50, 51), and Iran (28), which also observed rising mean DMFT scores with age. In our study, DMFT and its FT component were positively correlated with age, with the highest mean DMFT scores observed in the 41–50 year group. Several factors may explain these findings. Older individuals have had a longer cumulative exposure period, increasing their likelihood of developing caries. However, no significant associations were found between age and either DT or root caries scores. Moreover, although positive correlations were identified, the strength of association was weak (r < 0.5), lower than previously reported (28), and of limited clinical relevance.

The study has some limitations. The cross-sectional design only reveals associations between variables and cannot establish causal relationships. In this study, dental caries were primarily diagnosed through visual inspection. When necessary, a dental explorer was employed to verify lesion softness or detect enamel breakdown on proximal surfaces. One potential limitation is the absence of radiographic assessment, which may have led to underestimation of proximal caries. Such under-diagnosis could influence the study outcomes. However, previous research has indicated that radiographs add minimal additional value to epidemiological findings (30), suggesting that this limitation is unlikely to have had a substantial impact on our results.

Given the limitations of this research, longitudinal investigations are needed to clarify causal links between the identified risk factors and the occurrence of root caries, as well as factors influencing overall caries prevalence (DMFT). Furthermore, because the participants were exclusively from an Indian ethnic background, the applicability of these results to other racial and ethnic groups globally remains restricted.

The study was restricted to urban, middle-income participants from a single tertiary care hospital in Chandigarh, all brushing once daily, limiting its applicability to rural or diverse socioeconomic groups. Future studies can expand the sample to include rural and varied socioeconomic groups or explicitly state the urban focus as a limitation in the methodology. Excluding participants with systemic diseases or medications may omit those with conditions affecting caries risk (e.g., diabetes), potentially skewing prevalence estimates (52–56). Future studies can include and conduct a subgroup analysis for participants with common systemic conditions. The present study does not account for confounders like diet, fluoride exposure, education level of participants, or detailed oral hygiene practices beyond brushing frequency, which could influence caries outcomes. These can be addressed by collecting data on confounders and using multivariate analysis to adjust for their effects.

Provision of dental treatment to the ageing population in India will require an upgrade of the infrastructure of hospitals, more manpower with appropriate skills, and trained doctors and paramedical staff, adding more burdens to the health resources. Therefore, it is essential that an accurate assessment of oral health status be carried out in adults. On the basis of findings, appropriate early preventive intervention strategies should be developed and implemented. Greater emphasis should be on continuous prevention and promotion of oral health in the community for all age groups. In addition, clinicians and practitioners need to provide targeted chairside preventive measures and do a risk assessment besides treatment for all young adults, which currently is not given due attention.

## Conclusion

Findings of this study indicate that, as age increases, dental caries prevalence increases in this urban population. A significant difference exists in the prevalence of dental caries in the three adult age groups. Females had significantly more dental caries compared to males.

## Data Availability

The raw data supporting the conclusions of this article will be made available by the authors, without undue reservation.
